# Defensive Medical Practice in Dentistry: A Dual-Perspective Cross-Sectional Analysis of Dentists and Patients in Romania

**DOI:** 10.3390/healthcare14131992

**Published:** 2026-07-04

**Authors:** Ana Cernega, Marina Imre, Alexandra Ripszky, Bogdan Dimitriu, Vlad Gabriel Vasilescu, Silviu-Mirel Pițuru

**Affiliations:** 1 Department of Organization, Professional Legislation and Management of the Dental Office, Faculty of Dental Medicine, “Carol Davila” University of Medicine and Pharmacy, 17-23 Plevnei Street, 020021 Bucharest, Romania; silviu.pituru@umfcd.ro; 2 Department of Prosthodontics, Faculty of Dental Medicine, “Carol Davila” University of Medicine and Pharmacy, 17-23 Calea Plevnei, 010221 Bucharest, Romania; marina.imre@umfcd.ro; 3 Department of Biochemistry, Faculty of Dental Medicine, “Carol Davila” University of Medicine and Pharmacy, 17-23 Plevnei Street, 020021 Bucharest, Romania; alexandra.ripszky@umfcd.ro; 4 Department of Endodontics, Faculty of Dental Medicine, “Carol Davila” University of Medicine and Pharmacy, 17-23 Plevnei Street, 020021 Bucharest, Romania; bogdan.dimitriu@umfcd.ro; 5 Discipline of Dental Prosthesis Technology, Faculty of Dentistry, “Carol Davila” University of Medicine and Pharmacy, Dionisie Lupu Street, No. 37, District 2, 020021 Bucharest, Romania; vlad.vasilescu@umfcd.ro

**Keywords:** professional error, malpractice, doctor–patient relationship, defensive medical practice, VUCA, results pyramid

## Abstract

**Background**: Fear of malpractice and its potential legal, financial, and reputational consequences are associated with one of the most complex phenomena in the medical community: defensive medical practice (DMP). DMP is frequently analyzed in the specialized literature from the physician’s perspective; however, the patient’s role in triggering and maintaining defensive behaviors remains under-explored. **Methods**: This cross-sectional study examined contextual factors associated with fear of malpractice and the convergences between doctors’ and patients’ perspectives within a bilateral model (error–fear–perceived risk–prevention behaviors), without assuming direct causal relationship. Two questionnaires were administered in Romania to 240 dentists (March–June 2023) and 344 patients (June–December 2023). Associations were tested with chi-square and Fisher’s exact tests (reporting Cramér’s V and odds ratios), multivariate binary logistic regression, and post hoc power analysis. **Results**: Over half of dentists (53.3%) reported fear of malpractice despite minimal actual legal exposure (0.8%); this fear was associated with awareness of its potential consequences and perceiving patients as more demanding. In multivariate analysis, fear was the strongest independent predictor of perceiving patients as a threat (aOR = 3.98, 95% CI [1.67–9.48]). On the patient side, 57.9% would avoid a dentist with a known malpractice case and 34.0% had requested additional procedures for reassurance. **Conclusions**: The interaction between physician fear and patient pressure suggests the existence of a “reassurance loop”, in which the patient’s need for safety and the doctor’s fear can mutually reinforce each other, fostering defensive behaviors. We propose an exploratory typology of patient-induced DMP—direct induction (explicit requests for additional investigations/procedures) and indirect induction (relational pressure and reassurance seeking)—to guide future research. By integrating the dentist and patient perspectives within a bilateral model, the study provides a context-specific account of patient-induced defensive practice in Romanian dentistry and identifies dual-target educational interventions (addressing both clinician communication and patient health literacy) as a potential preventive direction.

## 1. Introduction

The effective management of the doctor–patient relationship has become one of the most pressing concerns in contemporary healthcare, shaped by a context often described through the VUCA framework—volatility, uncertainty, complexity, and ambiguity. Originally developed in strategic management to describe destabilizing forces in complex environments, the framework has been adopted in medicine to characterize the contextual pressures shaping contemporary professional practice [1,2,3,4,5,6,7]. In the medical setting, these manifest as fluctuating service costs [3,8], unpredictable legislative change [1,9], the influence of mass media [10,11,12] and of litigation-oriented legal practice [13,14,15], and uncertainty surrounding malpractice insurance [16,17]. Coping with this complexity requires continuous learning and the “*unlearning*” of outdated routines [18,19,20,21,22,23], while the doctor–patient relationship—the constitutive cell of the health system—remains under sustained strain.

The VUCA context exerts such tremendous pressure on the medical community that it can increase the risk of ***professional error*** [24], which, in turn, may damage this relationship [7]. In response, the patient, recognizing the harm caused, may file a malpractice claim to trigger the doctor’s legal liability. This mechanism, grounded in the right to compensation for potential damages, gives rise to the dimension of ***medical malpractice***—one of the most pressing and challenging consequences of VUCA.

The specialized literature points to a continuous growth in the dimension of malpractice, in the sense of an increasing number of court cases, as well as growing financial pressure on the culpable doctor through higher amounts paid as material and/or moral damages [25,26,27,28,29,30,31,32,33]. By becoming aware of the potential consequences of involvement in such legal proceedings, the medical community develops the “*clinical–judicial syndrome*” [34,35] and the “*medical malpractice stress syndrome*” [36], which would justify and push the doctor toward the need to react in order to protect themself from professional and reputational risk [37,38].

The doctor’s reaction materializes as a boomerang effect in the form of ***defensive medical practice*** (DMP), which turns back on the patient who initiated the legal procedure [39,40,41,42,43]. The DMP phenomenon develops as a result of the doctor being affected and victimized, who, in turn, will react in two ways:

▪ *Negative reaction*—the doctor will refuse to treat patients with a high medical risk or will refuse to perform specific medical procedures considered risky and likely to entail possible complications [16,43,44];
▪ *Positive reaction*—the doctor will unjustifiably recommend additional tests, in order to avoid conflict and minimize the risk of a potential malpractice accusation [40,43,45,46,47].

Many studies have examined the phenomenon of DMP globally, highlighting its significant impact on the doctor–patient relationship and the effective execution of medical acts [48,49,50,51]. Additionally, this phenomenon places considerable ethical, legislative, and economic pressure on the healthcare system [32,48,49,50,51,52].

Within this broader phenomenon, defensive practice in dentistry has only recently begun to be characterized as a distinct field of inquiry. Recent qualitative and questionnaire-based studies indicate that defensive behaviors are common among dental practitioners and are shaped by litigation anxiety, patient relational pressure, and the complexity of clinical decision-making [53,54,55,56]. Patient-side evidence points to the rising role of reassurance seeking and information-driven behavior in shaping the dentist–patient interaction [57]. The dental setting also differs from general medicine in important ways (frequent elective procedures, direct out-of-pocket payment in private practice, and a high reliance on patient cooperation) which may alter both the triggers and the manifestations of defensive practice.

Recognizing the complexity of this phenomenon, this research aims to map out the perceived factors and provide a descriptive analysis of the context that fosters litigation-related fear and defensive practices in Romania. To achieve this, we set out to extend our analysis by adopting a dual perspective on this issue. We will integrate two distinct dimensions and viewpoints that, while different, complement and mutually influence one another:

▪ An analysis of the factors specific to DMP among dentists in Romania.
▪ An analysis of the influence of patients in triggering and developing defensive behavior among dentists in Romania.

While most research on DMP focuses solely on the physician’s perspective, it is essential to understand this issue from both sides. As discussed, the phenomenon arises as a reaction to an action, highlighting the dynamic interaction between the dentist and the patient.

The study has four aims. We describe the contextual factors associated with fear of malpractice and DMP in the sampled dentist group. We look at patient behaviors that may induce or amplify defensive responses in dentists. We test associations between these factors using bivariate and multivariate models. And we propose a typology of patient-induced DMP for further empirical testing. The null hypothesis is that the contextual factors examined (demographic, experiential, relational) are not associated with indicators of defensive practice in either sample.

## 2. Materials and Methods

This study employed a cross-sectional design, using two questionnaires administered to two groups in Romania: dentists and patients. The dentists’ group consisted of 240 participants, while the patients’ group consisted of 344 participants. Participants were recruited through convenience sampling from Bucharest and its surrounding areas. The dentists were approached during their participation in professional training courses, while the patients were adult individuals who had received dental services and were recruited from dental practices. This recruitment route accounts for the urban predominance of the resulting sample; its implications for representativeness are addressed in Section 4.4. All questionnaires were fully completed.

The development of the questionnaires was prompted by a recurrent concern voiced by Romanian dentists during professional orientation, legislation, and management courses delivered by members of the research team—the contextual factors surrounding malpractice exposure and defensive practice in their everyday work. Drawing on this field-based input, on the team’s expertise in medical law and professional management, and on the existing literature on DMP and malpractice-related behaviors, the items were generated and subsequently adapted to the Romanian medico-legal context. Their wording was crafted to ensure clarity and consistent comprehension among respondents; adjustments were made to minimize ambiguity and to avoid language that presupposed advanced legal knowledge. Content validity was established through an iterative expert-review process within the research team, in which the items were appraised for conceptual coherence, clarity, and contextual relevance and refined accordingly. The dentist instrument contained 16 analyzed items and the patient instrument 13, with closed-ended responses (Yes/No/Don’t know) and multi-select options where applicable. Internal consistency of the resulting thematic item groups was subsequently examined using Cronbach’s α; the implications of the low coefficients obtained are addressed transparently in Section 4.4.

The questionnaires were organized into four sections: *general information*; *errors in professional activity*; *management of the doctor–patient relationship*; and *the need for caution in professional practice*.

The questionnaire for dentists was administered between March and June 2023, while the patients’ questionnaire was distributed from June to December 2023. Participants were briefed on the study’s objectives and protocols and were required to sign written informed consent before taking part. The questionnaires were administered on paper under conditions that ensured confidentiality, enabling anonymous participation. After collection, the responses were entered into a database designated for analysis, and processing was conducted solely on anonymized datasets.

Both study components received approval from the Scientific Research Ethics Committee of the “Carol Davila” University of Medicine and Pharmacy in Bucharest (approval no. 5734/24.02.2023) and were conducted in accordance with the recommendations of the Declaration of Helsinki and the good clinical practice guidelines.

Analyses were conducted using JASP version 0.19 (JASP Stats: Amsterdam, The Netherlands) [57]. Categorical variables are reported as counts and percentages. We compared groups with Pearson chi-square tests, and used Fisher’s exact test where expected counts fell below 5 in more than 20% of cells. Post hoc cell comparisons used Z-tests with Bonferroni correction. For chi-square tests we report Cramér’s V as the effect size; for 2 × 2 tables we also report odds ratios with 95% confidence intervals. We then fitted binary logistic regression models for two outcomes most relevant to the conceptual framework: fear of malpractice (D1) and perception of patients as a threat (D2). Predictors were set in advance based on the conceptual framework and covered demographics (age category, gender, sector of practice, specialty status) and psychosocial variables. We report adjusted odds ratios (aORs) with 95% CIs, and the likelihood-ratio test, McFadden’s pseudo-R^2^ and AIC for model fit. For descriptive tables we kept the three response categories (Yes, No, Don’t know). For the regressions, “Don’t know” responses were treated as missing (listwise deletion); sensitivity analyses with alternative codings produced consistent estimates. Cronbach’s α and mean inter-item correlations were calculated within thematic groups of items. The questionnaire was developed for content coverage rather than as a single unidimensional construct, and the low α values bear this out: we report them transparently. A post hoc power analysis was performed using G*Power 3.1 [58]. With N = 240 dentists we had 80% power to detect Cohen’s w ≥ 0.18 (df = 1) at α = 0.05; with N = 344 patients, w ≥ 0.15. Statistical significance was set at α = 0.05. Dentists and patients were analyzed as independent samples.

## 3. Results

### 3.1. Analysis of Factors Specific to DMP Among Dentists in Romania

According to Table 1, the dentist sample was distributed predominantly between the 35–65 (59.6%) and under-35 (34.6%) age categories, with respondents above 65 accounting for a marginal proportion. Practice was concentrated in the private sector (75.4%), and general dentists constituted the majority of respondents (73.7%), with the remainder holding a specialist title.

#### 3.1.1. Dentists’ Perception of Error in Professional Activity

**Figure 1:**
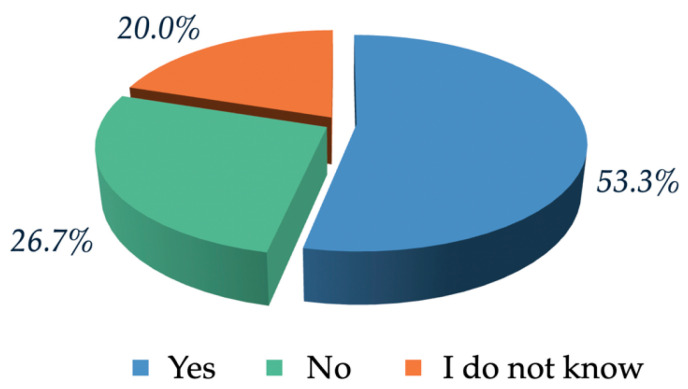
Distribution of dentists with respect to the existence of the feeling of fear.

Past medical errors were acknowledged by 46.25% of the dentists, with 24.58% denying such occurrences and 29.17% selecting the “*I do not know*” option. By contrast, only 0.8% of respondents had been involved in legal proceedings for the establishment of professional liability. Fear of malpractice was reported by 53.3% of the dentists, while 26.7% reported no such fear and 20.0% selected “I do not know”—a sizeable share of the sample positioned between the two response poles (see Figure 1).

**Figure 2:**
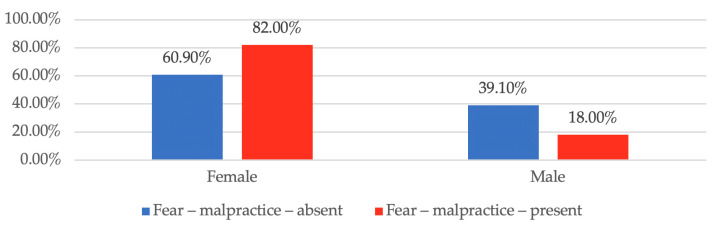
Distribution of dentists based on the presence of fear and gender.

Furthermore, according to Table 2 and Figure 2, female dentists reported fear of malpractice significantly more frequently than male dentists (82.0% vs. 60.9%; Fisher’s exact test, *p* = 0.002; OR = 2.93, 95% CI [1.49–5.75]; Cramér’s V = 0.23, indicating a medium-strength association; Table 2; Figure 2).

***Awareness of a colleague’s involvement in litigation*** was reported by 24.2% of dentists, while 11.3% remained uncertain and 64.5% reported no such awareness. When related to the responses regarding fear of malpractice, a pattern emerged: among the 64 respondents who reported no fear of being involved in a malpractice case, 68.8% also reported no awareness of colleagues in such proceedings, whereas among the 128 respondents who reported fear, 30.5% were aware of at least one colleague’s case. This pattern is consistent with the documented effect of vicarious litigation exposure on professional stress and the development of malpractice-related stress syndrome [59].

Most dentists (87.1%) considered that a patient filing a malpractice complaint could ***affect professional activity***, while 12.9% did not share this view.

***Confidence in malpractice insurance*** was mixed: 45.4% of dentists considered it effective, 20.8% did not, and 33.8% selected “I do not know,” reflecting uncertainty about the protection it offers. Among the 64 respondents who reported no fear of being involved in a malpractice case, 54.7% also reported confidence in the effectiveness of malpractice insurance, suggesting an inverse association between perceived insurance reliability and fear.

#### 3.1.2. Dentists’ Perception of the Management of the Doctor–Patient Relationship

**Figure 3:**
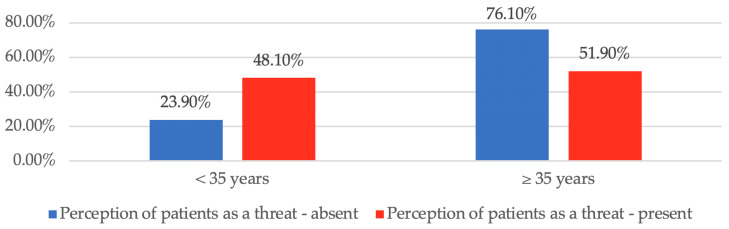
Distribution of dentists regarding patients’ perception as a threat and the participants’ age.

Perception of ***patients as a threat*** was reported by 33.75% of dentists, while 36.67% did not perceive them as such and 29.58% were unsure. Dentists under 35 reported perceiving patients as a threat significantly more often than those aged ≥ 35 (Fisher’s exact test, *p* = 0.001; OR = 2.92, 95% CI [1.51–5.63]; Cramér’s V = 0.25, indicating a medium-strength association), as shown in Table 3 and Figure 3.

At the same time, we find that 70% of dentists (168 respondents) have noticed that their ***patients have become more demanding***; 20.8% (50 respondents) chose the answer “no,” and 9.2% (22 respondents) are unsure about this. According to the Pearson Chi-Square test (*p* = 0.040), dentists aged ≥35 years, in light of their greater professional experience and larger patient portfolio, more frequently reported considering patients to be more demanding (69.6% vs. 54%) compared with dentists aged <35 years (46% vs. 30.4%), as shown in Table 4.

We also observed that dentists working in the private system more frequently reported that their patients had become more demanding (89.9% vs. 77.8%) than those in the public system (22.2% vs. 10.1%), according to Fisher’s exact test (*p* = 0.044).

#### 3.1.3. Dentists’ Perception of the Need for Caution in Professional Activity

The following subsection complements the dimension of defensive behavior within the target group by analyzing the type of behavior they might adopt.

A total of 82.1% of dentists (197 respondents) acknowledged that they had ***refused patients*** during their professional activity, while 17.9% (43 respondents) did not adopt such an attitude. More important than this aspect are the reasons underlying the refusal of patients:

▪ 132 dentists (67%) refused patients because the complexity of the medical case exceeded the professional’s competence limits;
▪ 121 dentists (61.4%) refused patients because they were difficult in terms of their manifested behavior, having unjustified expectations;
▪ 9 dentists (3.8%) refused patients due to fear of the possibility of legal liability being engaged;
▪ 7 dentists (3.6%) refused patients out of fear of making a mistake.

From a professional standpoint, it is both ethical and legal to refuse treatment of a patient if the complexity of the dental procedure exceeds one’s area of expertise.

At the same time, according to Table 5, the differences between groups are statistically significant: dentists aged ≥35 years significantly more often reported that they had refused patients (69% vs. 48.8%) compared with dentists <35 years (51.2% vs. 31%) (Fisher’s exact test, *p* = 0.012; OR = 2.34, 95% CI [1.20–4.57]; Cramér’s V = 0.16).

Regarding positive defensive practice, most dentists—82.9% (199 respondents—do not unjustifiably ***recommend additional procedures/tests***. However, 10% of dentists (24 respondents) acknowledge adopting such an attitude, and 7.1% (17 respondents) are uncertain about this. As for the reasons:

▪ 11 dentists (45.8%) recommend unjustified additional medical procedures to complex patients with unjustified expectations;
▪ 10 dentists (41.7%) recommend unjustified additional medical procedures at the explicit request of the patient;
▪ 3 dentists (12.5%) recommend unjustified additional medical procedures out of fear of making a mistake;
▪ 3 dentists (12.5%) recommend unjustified additional medical procedures out of fear of the possibility of legal liability being engaged.

### 3.2. Analysis of the Influence Exerted by Patients on the Triggering and Development of Defensive Behavior Among Dentists

According to Table 6, the vast majority of patients—76.2% (262 respondents)—are under 35 years of age; 23% (79 respondents) are between 35 and 65 years; and 0.8% (3 respondents) are over 65 years. A total of 71.2% of patients (245 respondents) are female, and 28.8% (99 respondents) are male. In total, 88.7% of patients (305 respondents) live in urban areas, while 11.3% (39 respondents) live in rural areas.

#### 3.2.1. Patients’ Perception of the Occurrence of Error in the Dentist’s Medical Activity

The first aspect analyzed within the target group refers to the ***identification of harm***. Approximately one in three patients (32.6%) reported having identified harm resulting from a possible error during a medical act, while 51.7% had not, and 15.7% were uncertain—a share that may correspond to harm perceived but not attributed to the dentist.

Patients’ reactions following the identification of harm followed the *avoidance, fight, and freeze* patterns of the four-mode framework [50,51]. The following distributions emerged:

▪ The majority adopted avoidance behaviors: 51.2% sought compensation through mediation, 35.5% turned to another dental practice, and 8.1% turned to another specialist within the same practice;
▪ A small proportion adopted typical fight behaviors: 4.4% requested formal sanctioning by the College of Dentists, 2.6% sought court-based sanctions, and 1.7% pursued compensation through a lawsuit;
▪ 3.5% froze and did not react.

In total, 57.85% of patients stated they would no longer use the services of a ***dentist known to have been involved in a court case***, 24.71% were uncertain, and 17.44% would continue. Male patients reported a significantly higher willingness than female patients to continue using a doctor with prior litigation (41.7% vs. 24.1%; Fisher’s exact test, *p* = 0.013).

Patients reported obtaining ***information about doctors’ legal involvement*** primarily through online news portals (74.4%), followed by social media (27%), television (20.3%), friends (18.6%), and family members (15.1%). Statistically significant differences emerged by age category (Fisher’s exact test, *p* < 0.05):

▪ Patients aged ≥35 years used television (38.6% vs. 20.1%, *p* = 0.003) and friends (35.9% vs. 21.1%, *p* = 0.015) as information sources significantly more often than younger patients;
▪ Patients under 35 used online news portals significantly more often than those aged ≥35 (79.3% vs. 67%, *p* = 0.029).

#### 3.2.2. Patients’ Perception of the Management of the Relationship with the Dentist

Recognizing the potential reputational risk that patients pose to a dentist’s professional activity, we aimed to identify the elements that contribute to an effective doctor–patient relationship, as well as factors that can disrupt its management. It is noteworthy that a significant majority of patients—96.8%—enjoy ***trusting relationships*** with their dentists. In contrast, 1.2% report a lack of trust, while 2% are unsure if trust exists in their relationship with their dentist.

Notably, 57% of patients ***recognize their ability to influence the treatment plan*** proposed by their dentist; 25.9% do not share this view, and 17.1% have no opinion, likely indicating a lack of concern about this issue. As illustrated in Table 7, statistically significant differences are observed among age groups, as determined by Fisher’s exact test (*p* < 0.001). Patients under 35 years of age are significantly more likely to feel they can influence their treatment plan compared to those 35 and older (87.2% vs. 59.6%).

One reason for this difference may be the younger generation’s willingness to utilize artificial intelligence tools, seek non-expert opinions, and consult multiple sources regarding proposed treatments. This influence can foster a positive perspective on the effectiveness of the relationship, emphasizing partnership and collaboration between the patient and the dentist, in line with the principle of autonomy. However, this dynamic can also create challenges. When patient influence becomes excessive, medical staff may feel pressured, complicating the doctor’s role as they must exert additional effort to reassure patients of their competence and skills.

Despite the high level of trust noted among patients, 55.2% still ***seek opinions from another doctor when they feel an acute need to confirm the diagnosis and recommended treatment of their initial dentist***. This is followed by 31.4% of patients who seek a second opinion after their initial treatment fails to meet expectations; 13.4% do so due to distrust in their dentist; 11.6% seek additional opinions in life-threatening situations; and 8.7% request a second opinion because of the absence of a multidisciplinary team at the first healthcare facility.

#### 3.2.3. Patients’ Perception of the Need for Caution in the Relationship with the Dentist

As defined at the outset, the present research aims to identify the current characteristics of the doctor–patient relationship and the patients’ behaviors that can prompt DMP among dentists.

Thus, from the perspective of the positive form of DMP, in terms of perception, 79.6% of patients (274 individuals) do not believe that their dentist has ***recommended medical procedures without justification and medical necessity***; 7.3% of patients (25 respondents) consider that their dentist adopts such attitudes; and 13.1% of patients (45 respondents) are unsure about this aspect. Nevertheless, it can be seen that patients constitute an important lever in prompting dentists to engage in defensive behavior, as 34% of patients (117 respondents) request that the dentist perform additional medical procedures. In this context, to please the patient and reduce conflict, the dentist will recommend or perform the requested procedures. A total of 63.4% of patients (218 respondents) have never requested additional procedures, and 2.6% of patients (9 respondents) do not know whether they have made such requests.

The main reasons for requesting the recommendation or performance of additional medical procedures are as follows: 38.5% of patients (45 respondents) need confirmation of the diagnosis and the recommended treatment methods; 26.5% of patients (31 respondents) state that the initial treatment did not produce the expected results; 21.4% of patients (25 respondents) have doubts or misunderstandings regarding the treatment; 10.3% of patients (12 respondents) report the existence of a life-threatening condition; 7.7% of patients (9 respondents) point to the absence of a multidisciplinary team; and 4.3% of patients (5 respondents) report a lack of trust in the dentist.

From the perspective of the negative form of DMP, we find that ***dentists have refused*** 17.1% (59) of patients in ways they perceived as unjustified, 81.7% (281) have not experienced such situations, and 1.2% (4) are unsure. Although we note a small number of refusals, when analyzing the reasons, 61% of patients (36) are refused because the medical case exceeds the dentist’s competence limits; 33.9% (20) were refused due to the dentist’s fear of making a mistake; and 10.2% (6) were refused because of fear of the possibility of legal liability being engaged.

### 3.3. Multivariate Analyses

To examine independent predictors after mutual adjustment, two binary logistic regression models were fitted, focusing on the outcomes most directly relevant to the conceptual framework: fear of malpractice (D1) and perception of patients as a threat (D4).

*Model D1*—fear of malpractice (N = 188 after listwise deletion of missing values; 128 reporting fear). After mutual adjustment for age, gender, sector of practice, specialty status, knowing colleagues involved in litigation, awareness of consequences, trust in malpractice insurance, and self-reported errors, male gender was the only predictor reaching conventional significance: men had substantially lower odds of fear compared with women (aOR = 0.34, 95% CI [0.16, 0.72], *p* < 0.01), corresponding to approximately 2.9-fold higher odds among female dentists. Awareness of the consequences of a malpractice accusation showed a marginal association in the expected direction (aOR = 2.54, 95% CI [0.94, 6.83], *p* = 0.06), as did lower trust in malpractice insurance (aOR = 0.53, 95% CI [0.26, 1.08], *p* = 0.08). Age, sector, specialty status, knowing colleagues involved in litigation, and self-reported errors were not independently associated with fear. The model fitted significantly better than the intercept-only model (LR χ^2^(9) = 24.31, *p* < 0.01; McFadden pseudo-R^2^ = 0.10; Nagelkerke R^2^ = 0.17).

*Model D2*—perception of patients as a threat (N = 142; 80 perceiving patients as a threat). Fear of malpractice was the strongest independent predictor (aOR = 3.98, 95% CI [1.67, 9.48], *p* < 0.01), consistent with the hypothesis that fear shapes how dentists construe the patient before any defensive action is taken. Three additional factors reached significance: younger age (aOR for ≥35 vs. <35 = 0.28, 95% CI [0.11, 0.69], *p* < 0.01; equivalently, ~3.6-fold higher odds among dentists under 35), female gender (aOR for male vs. female = 0.25, 95% CI [0.10, 0.64], *p* < 0.01), and knowing a colleague involved in litigation (aOR = 2.75, 95% CI [1.10, 6.85], *p* = 0.03). Specialists were significantly less likely than general practitioners to perceive patients as a threat (aOR = 0.32, 95% CI [0.13, 0.81], *p* = 0.02), a novel finding that may reflect greater professional confidence with specialization. Model fit was strong: LR χ^2^(7) = 44.62, *p* < 0.01; McFadden pseudo-R^2^ = 0.23; Nagelkerke R^2^ = 0.36.

## 4. Discussion

Throughout this section, we distinguish between empirical findings (the contextual factors and behavioral patterns directly observed in the data) and hypothesized mechanisms, including the bilateral conceptual model, the “*reassurance loop*”, and the proposed typology of patient-induced DMP. The latter constitute interpretive scaffolding generated from the present data, intended to organize the existing literature on DMP and to guide future research, rather than relationships causally tested in this study. This study is therefore best read as hypothesis-generating.

### 4.1. Summary of Main Results

**Figure 4:**
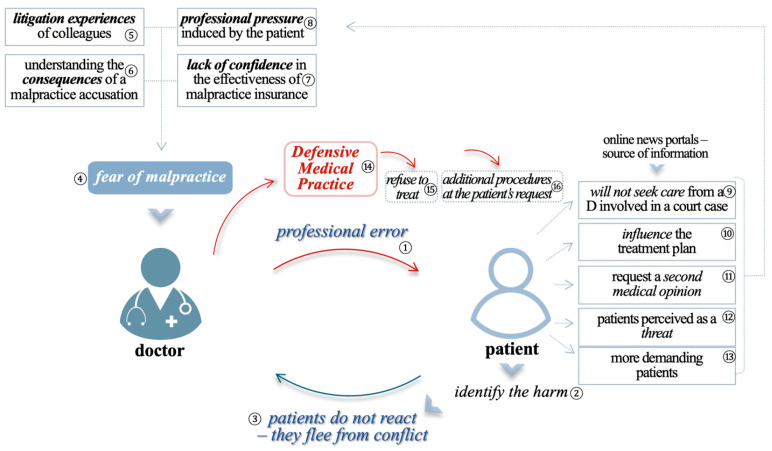
Conceptual map of defensive practice-related triggering factors in Romania—a dual-perspective view (dentist–patient).

To facilitate interpretation of the results and highlight the logic of the relationship between the variables investigated, Figure 4 schematically summarizes the bilateral dimension of the multitude of factors that may trigger the phenomenon of DMP in the context of dentistry in Romania.

The model does not imply a direct causal relationship between its elements; rather, it organizes the interactions between doctors’ and patients’ responses into a logical sequence: “*error–pressure–fear–perceived risk–prevention behaviors*”. Within this framework, the research highlights the prevalence of fear and examines the contextual factors that accompany it, in a manner consistent with the literature on DMP.

#### 4.1.1. Professional Error—A “Core” Vulnerability

At the centre of the model lies professional error ①, around which the reactions of both parties are organized. A notable share of dentists were uncertain when self-assessing their own errors, a pattern that suggests limited error awareness and may itself sustain a posture of defensive caution. On the patient’s side, the model begins with the identification of harm ②. Although harm recognition might be expected to prompt a legal response, patients overwhelmingly preferred conflict avoidance ③ and amicable resolution over litigation—consistent with the very low incidence of actual legal exposure reported by dentists. This tendency is supported by the doctors’ responses, as the overwhelming majority stated that they had not been involved in any legal proceedings.

This discrepancy between the perceived frequency of errors in medical practice and the low actual incidence of legal exposure is crucial for understanding the unique characteristics of this phenomenon in Romania. Consequently, the medical community perceives the legal threat as a possibility, even if it has not been directly experienced [60,61,62,63]. This situation means that the doctor’s fear remains unvalidated by legal action but does not dissipate; it continues to be a potential concern for the future.

#### 4.1.2. Fear of Malpractice—The Driver of Defensive Behaviors

In a context of significant uncertainty, fear of malpractice emerges as a primary motivator for professional responses [60,61,62], reported by over half of the dentists despite minimal direct legal exposure. Several factors amplify and reinforce this fear:

▪ *Litigation Experiences of Colleagues*: awareness of peers who have faced legal proceedings points to a mechanism of social risk transmission, in which fear is heightened by anticipated rather than directly experienced consequences [38,56,64].
▪ *Understanding the Consequences of a Malpractice Accusation:* the near-universal recognition among dentists of the serious implications of a malpractice claim supports the idea that fear operates as an “interface” between potential error and defensive practice, aligning with the documented “clinical–judicial” and “malpractice stress” syndromes [34,35,36,37,38,56].
▪ *Lack of Confidence in Malpractice Insurance Effectiveness:* although legally mandatory, the policy is often perceived as a licensing formality, and limited familiarity with its exclusions and contractual scope sustains mistrust. This pervasive uncertainty about the protective value of insurance appears to exacerbate fear and reinforce defensive tendencies [16,65].
▪ *Media Exposure:* The prominence of malpractice cases in mass media can amplify fear by undermining the professional’s reputational security and by foregrounding the rational and emotional repercussions of an accusation (loss of time, money, patient portfolio, suspension of activity, and physical and psychological harm).

#### 4.1.3. Professional Pressure Induced by Patients

The framework also highlights that professional pressure from patients ⑧ plays a critical role in the development of litigation-related fear. This pressure isn’t always articulated as a direct accusation; instead, it manifests as relational and decisional pressure. Patients may avoid consulting a doctor perceived to have been involved in a legal case ⑨, influence the treatment plan ⑩ [64], or request second opinions to confirm diagnoses ⑪ [66].

From the doctor’s perspective, this pressure is reflected in various patient behaviors, where patients are viewed as potential threats ⑫ and exhibit heightened demands ⑬ [66]. This dynamic results in a more complex doctor–patient relationship, which tends to be more negotiated and sensitive to perceived risks.

These pressure dynamics did not unfold uniformly across all participants. Younger dentists appeared less confident in their professional activity and in managing patient relationships, which may translate into a more cautious stance shaped by limited experience, fear of error, and communication difficulties. Among patients, gender moderated the willingness to engage with a clinician’s litigation history: male patients were more likely to continue using the services of a doctor previously involved in a malpractice case, consistent with research on gender differences in health-seeking behavior, where men tend to adopt a more assertive stance toward providers with prior litigation. Threat perception itself showed similar contextual sensitivity: dentists who did not perceive patients as a threat appeared to rely on professional confidence and on the absence of prior litigation or harm-identification experiences, while the sizeable undecided group points to latent ambivalence rather than firm reassurance.

Beyond participant characteristics, structural features of the care setting also shape how this pressure is experienced. Dentists in private practice more frequently reported that their patients had become demanding than those in the public sector, a difference plausibly linked to how dental care is financed in Romania: national legislation regulates the partial or full reimbursement of a defined set of therapeutic procedures, with patients contributing via special co-payment, while in the private sector the cost falls entirely on the beneficiary. Direct payment may raise patients’ expectations and translate into more explicit demands on the clinician.

#### 4.1.4. DMP—Behavioral Reactions

The interplay among fear ④, mistrust in instruments ⑦, and patient pressure ⑧ might culminate in DMP ⑭ [53,54]. Defensive practices are professional responses aimed at mitigating the perceived risk of potential accusations, particularly in environments where legal protections seem incomplete or uncertain. This research identifies two visible forms of DMP:

▪ *Refusal to Treat* ⑮: This negative or avoidant defensive behavior is illustrated by reports from doctors who refuse patients, as well as patients’ experiences of being refused care [16,41,42,43,44,56].
▪ *Recommendation of Additional Procedures* ⑯: This active defensive behavior occurs when doctors, prompted by patient requests for additional procedures, may recommend more tests or interventions, especially in areas of uncertainty or under decisional pressure.

It is important to note that not every refusal of treatment or recommendation of additional diagnostic procedures constitutes defensive practice. Refusing a case that genuinely exceeds one’s professional competence or seeking diagnostic confirmation in clinically uncertain situations are appropriate components of professional risk management. Defensive practice is distinguished by the primary motivation of self-protection from legal or reputational consequences, even when this motivation overrides or substitutes for clinical judgment. The empirical data presented here support this distinction: while a majority of dentists reported having refused patients (82.1%) or, less frequently, having recommended additional procedures (10.0%), only a small proportion attributed these behaviors specifically to fear of legal liability (3.8% of refusals; 12.5% of additional procedures) or fear of error (3.6% of refusals; 12.5% of additional procedures). The remaining majority of these behaviors were attributed to clinical or relational factors that fall within legitimate professional conduct.

### 4.2. Proposed Exploratory Typology: Passive vs. Active Induction of DMP

Building on the results of this study and the role patients play in triggering DMP, we propose an exploratory typology, intended as a hypothesis-generating framework rather than a validated classification, that complements the traditional positive/negative classifications of DMP. This typology focuses on how patients influence medical decision-making.

▪ *Passive (Indirect) Induction:* This refers to the background influence exerted by patients through factors such as mistrust, uncertainty, ambiguous information (often from media sources), unrealistic expectations, and the need for reassurance. These elements can increase the pressure felt by doctors, even in the absence of an explicit request from the patient. In the present sample, passive induction is reflected in patterns such as the 55.2% of patients who sought a second opinion, the high reliance on online news portals as a source of information about doctors, and the 57.85% who would avoid a dentist with a known malpractice case.
▪ *Active (Direct) Induction:* This involves patients making explicit requests for additional investigations, consultations, or medical procedures. In this case, patients play a role in confirming and validating the need for such actions. These requests may lead to positive defensive recommendations—that is, doctors may opt to “*do more to be safer*” to minimize the risk of potential conflicts with patients. Active induction, in the present sample, is operationalized through the 34.0% of patients who reported having requested additional procedures from their dentist.

This typology does not replace the existing positive/negative classification of DMP. Instead, it highlights a potential source of defensive practice, namely, the patient, who can amplify the situation through both indirect influences and direct requests. Thus, the study’s findings suggest that it is not solely the fear of malpractice that may drive additional procedures (positive defensive practice). The patient’s need for reassurance can also contribute to an increased decision-making burden, resulting in additional investigations and procedures.

Furthermore, these requests for reassurance such as seeking second opinions or requesting further investigations can create a dangerous cycle: (1) the patient asks for confirmation; (2) the doctor recommends additional measures (positive defensive practice); (3) the patient interprets “*more*” *as* “*safer*”; (4) the likelihood of future requests increases, intensifying the pressure on the doctor and the healthcare system as a whole [32,43,48,49,50,51,52].

### 4.3. Individual and Systemic Implications

The present research starts from a professional context characterized by volatility, uncertainty, complexity, and ambiguity (VUCA), which, through the pressure it exerts, can increase the risk of professional error, amplify the perceived risk of malpractice, and favor DMP [7,67]. Within this framework, a practical strategy is required to serve as a remedy, through interventions aimed at the beliefs and actions of the involved parties.

In the logic of the proposed bilateral model (doctor–patient), the results suggest a circuit of reactions that can function as a “*reassurance loop*”, in which the patient’s need for safety and the doctor’s fear can feed on each other, driving defensive behaviors. In this context, it is essential to define a strategy that can help neutralize existing behaviors and, more importantly, prevent them in the future.

**Figure 5:**
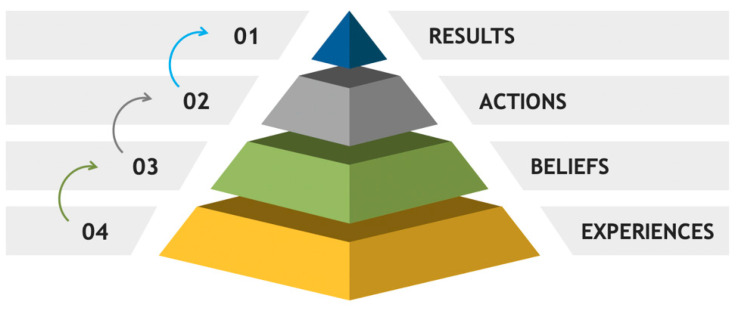
The “Results Pyramid” defined by Roger Connors and Tom Smith.

The “*Results Pyramid*” defined by Roger Connors and Tom Smith is a tool that can support such a change-management approach by modifying the pyramid’s base, which generates cascading changes at the other levels [68], as shown in Figure 5.

In the current context, the base of this pyramid is the VUCA phenomenon in healthcare—a difficult-to-change environment. Thus, the proposed strategy should not aim to eliminate the existing context, but rather to recalibrate the beliefs of system actors so that these beliefs lead to different actions and safer outcomes.

The implications at the individual level concern the two actors of the constitutive cell:

▪ ***Patients*** need medical literacy to comprehend the complexities of medical procedures [69,70] and the human factors involved in providing care, including the potential for human error [71]. Without this understanding, patients may become trapped in a reassurance loop, leading to repeated requests for confirmation and increased relational pressure. This situation can amplify defensive behaviors among healthcare providers [72,73]. This information dimension has been reshaped by patients’ growing use of artificial intelligence tools, non-expert opinions, and multiple online sources, consistent with the age gap observed in our sample (87.2% vs. 59.6% in those under 35 vs. 35 and older). When such influence becomes excessive it tends to feed the same loop, with the clinician working harder to reassure and reassurance itself sliding into defensive practice.
▪ It is essential for ***doctors*** to recognize the primary psychological need that patients present, which is the need for safety [74]. Additionally, doctors should focus on developing transversal competencies that are crucial for addressing patients’ needs and enhancing the doctor–patient relationship [75,76,77]. These two requirements converge in the informed-consent process, which should be treated not as an administrative formality but as a genuine instrument of the care relationship—the point where the patient’s understanding of risk and the physician’s communication meet, protecting the practitioner against unfounded claims while ensuring that patients grasp the risks involved [30]. The defensive behaviors observed in this study, together with the widespread distrust of malpractice insurance, suggest that this instrument is not yet perceived as dependable; clarifying its legal standing and training practitioners in its proper use could strengthen both actors’ position and reduce reliance on defensive responses.

In this pyramid logic, a change in the beliefs of both actors would have direct systemic implications. The outcome of this change would be the shaping of an ecosystem that offers safety and access to medical care, as well as professional security for the doctor, limiting the potential consequences for the system, particularly the high costs associated with DMP [32,43,48,49,50,51,52].

### 4.4. Limitations and Future Directions

Several limitations should be considered when interpreting these findings. The cross-sectional design permits description of how variables co-occur but not causal inference; we cannot establish that fear precedes the perception of patients as a threat, or that error precedes pressure. The sequence “*error → pressure → fear → perceived risk → behaviors*” is therefore best read as a heuristic organization of the existing literature rather than a causal structure tested in the present study.

Recruitment relied on convenience sampling as dentists were approached during professional training courses and patients through dental practices in a limited number of locations. This may introduce systematic bias, since dentists who attend continuing education may differ from non-attendees in conscientiousness and risk awareness. The findings cannot, therefore, be assumed to represent the broader Romanian dental population.

The instrument was developed for content coverage rather than as a unidimensional psychometric scale, and internal consistency across the thematic item groups was correspondingly low (Cronbach’s α = −0.18 to 0.33), as expected for items designed to capture distinct contextual factors. The negative coefficient for the “caution behaviors” group is itself informative, as willingness to report errors is conceptually opposed to defensive avoidance, so combining these items into a single scale leads them to counteract one another. Constructs such as fear, perceived patient threat, and reassurance pressure would benefit from dedicated, validated multi-item scales in future work.

The reliance on self-report introduces a further consideration, as sensitive items such as the acknowledgment of past errors or the recommendation of unjustified procedures are prone to under-reporting; the true prevalence of negative defensive behaviors is therefore likely higher than observed. In addition, because the two samples were independent and not paired at the consultation level, patients did not report on the dentists surveyed, and the proposed reassurance loop cannot be tested directly, this would require dyadic data from both parties to the same encounter.

Although statistical power was adequate for medium-to-large effects, it was limited (56–71%) for two associations with small effect sizes (Cramér’s V ≈ 0.14–0.16), which should be interpreted as suggestive; analyses based on very small cells (dentists over 65, n = 14; rural patients, n = 39) carry comparable caveats.

The associations reported in the present manuscript are based on individual items as outcomes (such as fear of malpractice, perception of patients as a threat, and refusal to treat), rather than on composite scale scores derived from these thematic groups, and the low α values therefore do not affect the validity of the bivariate and multivariate point estimates.

The substantial share of “I do not know” responses on several items constitutes a current limitation, since the present design cannot directly probe the meaning of these responses. At the same time, this pattern reinforces one of the central observations of the work—the marked uncertainty present in both samples.

As the first dual-perspective mapping of defensive medical practice in Romanian dentistry, the present design used general categories to capture the overall contours of the phenomenon, without distinguishing among the dental specialties or types of intervention involved.

Future research should pair dentist and patient data at the consultation level; use procedure-typed instruments stratified by dental specialty and intervention type; incorporate validated multi-item scales for the principal constructs (fear, perceived threat, patient pressure), including a validated index of patient pressure distinguishing passive from active induction; and complement closed-ended items with mixed-methods components capable of probing the meaning of “I do not know” responses. Structural-equation modeling would be a natural next analytical step. On the applied side, interventions targeting professional communication and the management of reassurance-seeking requests appear to offer the greatest potential benefit.

## 5. Conclusions

In this dual-perspective study, defensive medical practice in Romanian dentistry emerged as a bilateral dynamic in which the dentist’s fear of malpractice and the patient’s need for reassurance reinforce each other, sustaining a self-perpetuating reassurance loop. Fear was widespread among dentists and was independently associated with female sex, with prior or vicarious litigation exposure, and with the perception of patients as a threat. Among patients, harm recognition rarely translated into legal action, while younger patients more often reported the ability to influence their treatment plan. At the system level, this loop amplifies decisional pressure on clinicians and sustains both over-recommendation and refusal as defensive responses. The findings provide empirical grounding for the proposed distinction between active and passive forms of patient-induced defensive practice. Given the regional sample and non-paired design, these conclusions are exploratory and context-specific, requiring confirmation in larger, paired samples drawn from a broader geographic range; nonetheless, they offer an empirically grounded basis for interventions targeting both physician fear and patient health literacy.

## Figures and Tables

**Table 1:** General Information on the Dentists Participating in the Study.

Characteristics		Number	Percentage (%)
***Age category***	<35 years	83	34.6%
	35–65 years	143	59.6%
	>65 years	14	5.8%
***Gender***	female	182	75.8%
	male	58	24.2%
***Place of practice***	private system	181	75.4%
	public system	32	13.3%
	private + public system	27	11.3%
***Professional status***	general dentist	177	73.7%
	specialist dentist	63	26.3%

**Table 2:** Distribution of dentists based on the presence of fear and gender.

Gender/Fear—Malpractice	Absent		Present		*p* *	Cramér’s V	OR [95% CI]
	Nr.	%	Nr.	%			
**Female**	39	60.9%	105	82%	**0.002**	**0.23**	**2.93 [1.49–5.75] ^†^** **1.00 (ref)**
**Male**	25	39.1%	23	18%			

* Fisher’s Exact Test; ^†^ Odds ratio for female vs. male dentists. Female dentists had approximately 2.9-fold higher odds of reporting fear of malpractice (Cramér’s V = 0.23 indicates a medium effect).

**Table 3:** Distribution of dentists regarding patients’ perception as a threat and the participants’ age.

Age/Perception of Patients as a Threat	Absent		Present		*p* *	Cramér’s V	OR [95% CI]
	Nr.	%	Nr.	%			
**<35 years**	21	24.1%	39	48.1%	**0.001**	**0.25**	2.92 [1.51–5.63] ^†^ 1.00 (ref)
**≥35 years**	66	75.9%	42	51.9%			

* Fisher’s Exact Test; ^†^ Odds ratio for dentists under 35 vs. those aged 35 or older. Younger dentists had approximately 2.9-fold higher odds of perceiving patients as a threat (Cramér’s V = 0.25 indicates a medium effect).

**Table 4:** Distribution of dentists based on their perception of increasing patient demands and the age of participants.

Age/Perception of Patients’ Increased Demands	Absent		Present		*p* *	Cramér’s V	OR [95% CI]
	Nr.	%	Nr.	%			
**<35 years**	23	46%	50	29.9%	**0.040**	**0.14**	1.99 [1.04–3.82] ^†^ 1.00 (ref)
**≥35 years**	27	54%	117	70.1%			

* Pearson Chi-Square Test; ^†^ Odds ratio for dentists aged 35 or older vs. those under 35. Older dentists had approximately twice the odds of reporting that patients had become more demanding (Cramér’s V = 0.14 indicates a small-to-medium effect).

**Table 5:** Distribution of dentists based on their refusal of patients and the age of participants.

Age/Refusal to Treat	Absent		Present		*p* *	Cramér’s V	OR [95% CI]
	Nr.	%	Nr.	%			
**<35 years**	22	51.2%	61	31%	**0.014**	**0.16**	2.34 [1.20–4.57] ^†^ 1.00 (ref)
**≥35 years**	21	48.8%	136	69%			

* Fisher’s Exact Test; ^†^ Odds ratio for dentists aged 35 or older vs. those under 35. Older dentists had more than twice the odds of having refused at least one patient (Cramér’s V = 0.16).

**Table 6:** General characteristics of the target group—patients.

Characteristics		Number	Percentage (%)
***Age category***	<35 years	262	76.2%
	35–65 years	79	23%
	>65 years	3	0.8%
***Gender***	female	245	71.2%
	male	99	28.8%
***Place of residence***	rural	39	11.3%
	urban	305	88.7%

**Table 7:** Distribution of patients by treatment influence and participants’ age.

Age/Treatment Influence	Negative		Affirmative		*p* *	Cramér’s V	OR [95% CI]
	Nr.	%	Nr.	%			
**<35 years**	53	59.6%	171	87.2%	**<0.001**	***0.31***	4.66 [2.56–8.41] ^†^ 1.00 (ref)
**≥35 years**	36	40.4%	25	12.8%			

* Fisher’s Exact Test; ^†^ Odds ratio for patients under 35 vs. those aged 35 or older. Younger patients had approximately 4.7-fold higher odds of feeling they could influence their treatment plan (Cramér’s V = 0.31 indicates a medium-to-large effect).

## Data Availability

The data presented in this study are available on request from the corresponding author. The datasets are not publicly available due to participant privacy and confidentiality considerations.
